# Curated Collections for Clinician Educators: Five Key Papers on Graduated Responsibility in Residency Education

**DOI:** 10.7759/cureus.4383

**Published:** 2019-04-04

**Authors:** Benjamin H Schnapp, Holly A Caretta-Weyer, Eric Cortez, Scott A Heinrich, Aaron S Kraut, Christopher M Lloyd, Carly Silvester, Randy M Sorge, Amy Wain, Michael Gottlieb

**Affiliations:** 1 Emergency Medicine, University of Wisconsin, Madison, USA; 2 Emergency Medicine, Stanford University, Palo Alto, USA; 3 Emergency Medicine, Ohio University Heritage College of Osteopathic Medicine, Columbus, USA; 4 Emergency Medicine, Rush University Medical Center, Chicago, USA; 5 Emergency Medicine, Wide Bay Hospital and Health Service, Hervey Bay, AUS; 6 Emergency Medicine, Louisiana State University Health Sciences Center, New Orleans, USA

**Keywords:** graded responsibility, progressive responsibility, entrustable, epa, residency, gme, cbme, competency, housestaff

## Abstract

Introduction

The Accreditation Council for Graduate Medical Education calls graduated responsibility “one of the core tenets of American graduate medical education.” However, there is no clear set of resources for programs to implement a system of progressively increasing responsibilities for trainees. This project aimed to identify a set of high-yield papers on graduated responsibility for junior faculty members.

Methods

A study group of Academic Life in Emergency Medicine Faculty Incubator participants identified relevant literature on graduated responsibility via a comprehensive literature search and a call to the online medical education community; 59 total papers were identified. The most relevant and applicable were selected by the study group via a three-round modified Delphi process.

Results

Five key articles for junior faculty interested in implementing more robust graduated responsibility at their residency training program were selected and described here. Summaries of key points, along with considerations for faculty developers and relevance to junior faculty, are presented for each article.

Conclusions

The articles presented here provide a solid theoretical and practical basis for junior faculty to explore graduated responsibility. The five articles presented here provide the junior faculty with a toolkit to examine and improve their systems for assigning responsibilities in a graded fashion at their own institutions.

## Introduction

The responsibilities of an independently practicing physician are numerous. Beyond just seeing patients, a doctor may be expected to coordinate care with nursing and technician staff in their department, discuss pertinent results with radiology and laboratory staff, appropriately involve physicians from other medical specialties in the care of their patients, supervise learners and advanced practice providers, and troubleshoot problems with patient care as they occur in real-time. To the freshly minted doctor just out of medical school, taking on all of these responsibilities at once would be overwhelming. Therefore, training during residency must gradually allow the trainee physician to assume progressively more responsibility until they are ready to take on the mantle of independent practice themselves.

The Accreditation Council for Graduate Medical Education (ACGME) calls graduated responsibility “one of the core tenets of American graduate medical education” [1]. However, there is little guidance for training programs on how this can be best implemented, leaving programs to decide for themselves how best to layer on additional tasks and roles for trainees as they see fit. Many programs award this additional responsibility based on years of experience; a trainee with several years of experience takes on a more diverse set of tasks than a brand-new intern. While intuitively appealing, this structure overlooks individual differences in the speed at which proficiency develops. While one resident may take naturally to supervising junior clinicians, another resident at the same level of training may need significantly more practice.

Over the last few years, there has been an increasing push toward competency-based medical education (CBME), allowing trainee physicians to progress to independent practice when they demonstrate they are capable, rather than when their experience dictates they should. While this is one method of implementing graduated responsibility and represents an improved conceptual model over a strictly time-based progression of responsibilities, a competency-based system does not come with a user’s manual for implementation and programs interested in moving towards this model may feel limited by the lack of a clear way to move forward.

Currently, there is no collection of literature that exists to point the way for programs interested in improving or redefining their approach to graduated responsibility. The goal of this paper was to identify the most relevant and high-yield literature to aid educators in the implementation of graduated responsibility. To make this guide as effective as possible, included with each article is a brief summary, describing its utility for junior faculty and considerations for faculty developers.

## Materials and methods

The Academic Life in Emergency Medicine (ALiEM) Faculty Incubator is a year-long faculty development program focused on experiential learning and developing an online community of practice [2]. Within this program, several scholarship groups were formed to focus on topics of interest in medical education. The authors of this article selected graduated responsibility as their topic.

The study group consisted of six core members of the 2018-2019 ALiEM Faculty Incubator group. In order to incorporate broader perspectives, four additional participants external to the group with an interest in graduated responsibility were also identified and included. The final group included seven men and three women practicing Emergency Medicine in the United States and Australia. The years of clinical experience of the faculty in the group ranged from zero to 11 years.

**Figure 1:**
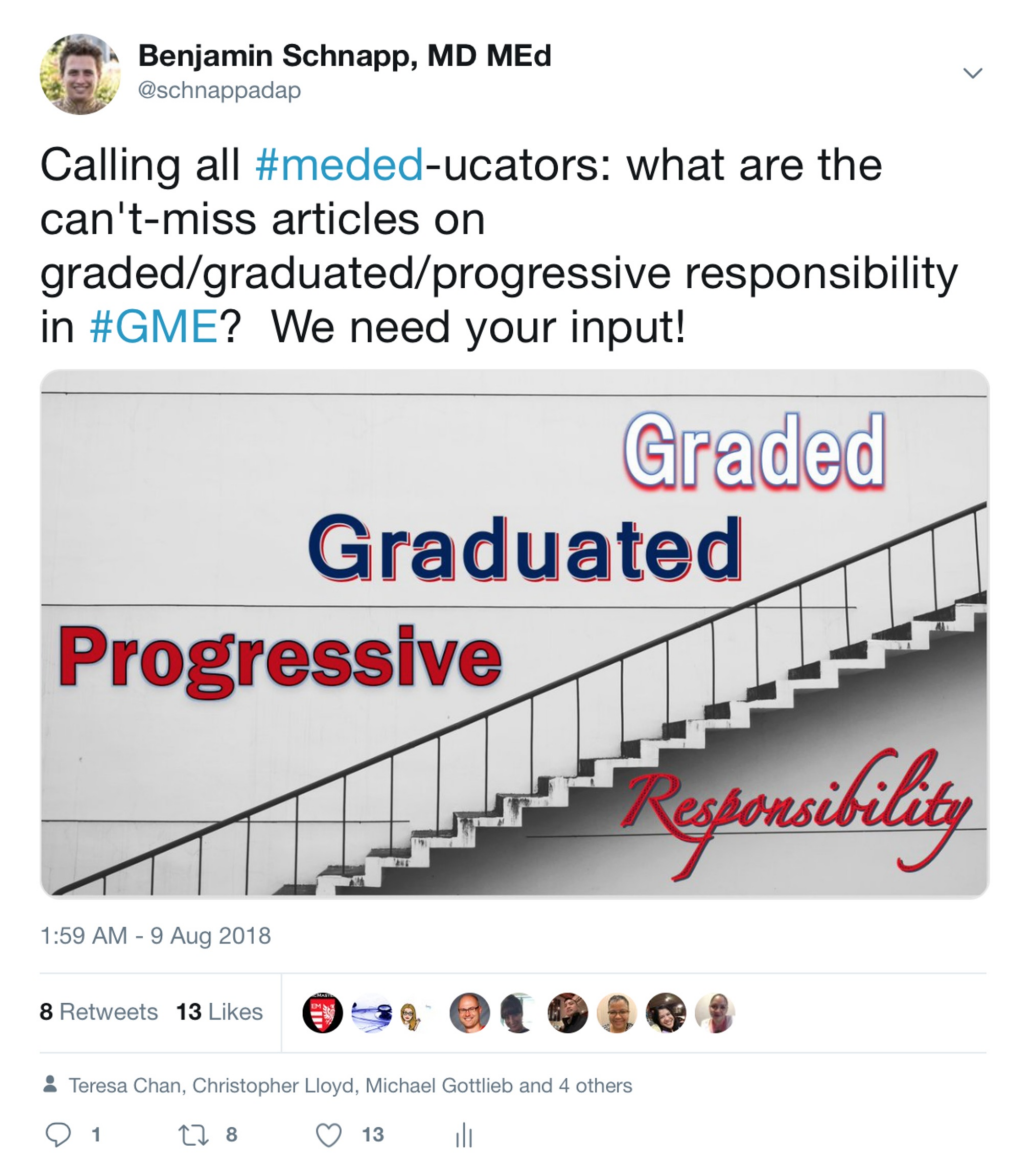
Solicitation to the online medical education community to submit relevant articles via a Twitter message

To identify relevant articles, five group members each undertook independent literature searches at their own institutions using PubMed and Google Scholar with the assistance of a medical librarian including search terms such as "graded," "progressive," "graduated," "responsibility," "entrustability," "independence," "supervision," "GME," "residency," and "housestaff.” To further ensure no relevant literature was missed, a call for articles was also placed to the medical education community on Twitter using the #meded hashtag (Figure 1).

This project was structured using a modified Delphi approach to select the final articles for inclusion in this guide. Unlike a true Delphi method (which uses only expert raters), we used junior faculty members as well, as it has been shown that experts may choose articles that do not fully meet the needs of more junior faculty [3]. A similar approach has been used in prior publications in this series [4-16].

During the first round of voting, participants read each of the articles and scored each on a Likert scale from 1 to 7, with one representing “No relevance for junior faculty interested in implementing graduated responsibility” and seven representing “Essential for junior faculty interested in implementing graduated responsibility.” Votes from round one were then compiled and the distribution of scores was shared with participants in round two. During round two, participants were asked whether each article should be included. Participants were allowed to choose more than five articles in this round. Votes from round two were compiled and the percentage of participants who thought each article should be included was shared with participants for round three. Finally, in round three, participants were asked to choose only the five articles that they thought were most relevant to be included in the manuscript. Five articles were chosen by group consensus as a reasonable number to adequately introduce junior faculty to the topic without being overwhelming.

## Results

A total of 59 unique articles were identified during the literature review process. Of these, 58 were identified via a web-based search; one additional article was identified using our Twitter query, which was viewed 3414 times. The Delphi process identified five articles (listed in the Discussion) as most critical for junior faculty interested in improving their residency’s approach to graduated responsibility.

## Discussion

Key papers

*1. Franzone JM, Kennedy BC, Merritt H, Casey JT, Austin MC, Daskivich TJ: Progressive Independence in Clinical Training: Perspectives of a National, Multispecialty Panel of Residents and Fellows. J Grad Med Educ. 2015, 7:700-704 *[17]

Summary: In this paper, the authors assembled a panel of residents and fellows from various medical specialties to identify factors that interfere with resident independence and to propose better structures for fostering progressive independence. The panel identified several themes, including poor feedback and mentorship, unevenly distributed supervision throughout training, and a lack of formal faculty development. To improve the current system, the panel recommended developing a well-structured curriculum focused on stepwise learning, pairing it with robust faculty development sessions and active mentorship. In order to provide objective assessments, the panel suggested creating additional educational milestones for leadership, teaching, and independent practice, allowing faculty to feel confident in functioning autonomously at these tasks when appropriate. There was also recognition that the public perception of trainees working independently is a complex problem that would likely require a collective approach aimed at addressing the legal, administrative, and financial factors, as well as creating incentives to accomplish these goals.

Relevance to junior faculty members: Although this paper identifies multiple factors that influence the current state of graduated responsibility in residency, two improvements appear to be most easily accomplishable and have the most relevance to junior faculty members: faculty development and two-way feedback. Faculty development sessions provide the foundational skills necessary to create more accurate and objective assessments of trainees’ abilities and two-way feedback creates a channel for faculty and trainees to clearly communicate their expectations and increase their comfort with fostering autonomous practice. Because junior faculty are still in the process of honing their craft, they have the unique opportunity to create good habits early on in their career. If junior faculty seek out faculty develop programs and commit to providing robust feedback as well as being willing to receive it, they may serve as a powerful model of behavior that may contribute to meaningful change on a broader level for trainees.

Considerations for faculty developers: This paper provides faculty developers with a broad insight into the challenges of implementing a graduated responsibility program from a resident perspective and promotes strategies for how they may be addressed. The diverse representation of residents included on the panel allows the consensus themes to be interpretable and implementable for faculty developers from various backgrounds and specialties. Two key themes that emerge from this paper, faculty development and two-way feedback, provide a focus for program improvement. However, the importance of generating ideas locally, as well as incentivizing and recognizing successfully implemented strategies, is also emphasized.

*2. Carraccio C, Englander R, Holmboe ES, Kogan JR: Driving Care Quality: Aligning Trainee Assessment and Supervision Through Practical Application of Entrustable Professional Activities, Competencies, and Milestones. Acad Med. 2016, 91:199-203 *[18]

Summary: This paper provides a framework for the practical application of the Accountable Assessment for Quality Care and Supervision (AAQCS) equation as defined by Kogan et al. [19]. The AAQCS equation suggests that safe, effective, patient-centered care results from the product of appropriate supervision matched to the individual trainee’s level of performance. Several challenges to achieving this matching are discussed: assessment, inter-rater variability, and appropriate clinical supervision for the developmental level of the trainee. A brief literature review is provided to equip faculty with tools to minimize these barriers. Group faculty development and proficiency with supervision skills are encouraged to enable a shared mental model and increase the accuracy of assessments. Global assessments are promoted over the use of checklists, as well as encouraging the variability of narratives from different assessors and adequate sampling to ensure the reliability of trainee assessments. The application of the AAQCS equation in the workplace is framed around entrustable professional activities (EPAs), core competencies, and specialty milestones. EPAs assist faculty with making informed decisions regarding assigning graduated responsibility, as assessors rate what level of supervision the trainee requires for each activity. The paper suggests that mapping EPAs to essential competencies allows the development of a shared mental model for supervisors of what a novice or advanced trainee would look like when engaged in a defined professional activity and allows more informed decisions on graduated responsibility and more effective feedback, in turn promoting quality care. 

Relevance to junior faculty members: Facilitating the advancement of medical residents from novice to expert while assigning graduated responsibilities is a complex process involving the recognition of the interdependence between learner and supervisor. The act of balancing this interdependent relationship can be difficult to traverse for both junior and senior faculty members. Junior faculty may benefit from understanding the challenges this may pose within a clinical learning environment. This manuscript provides junior faculty with an excellent introduction to the principles of EPAs and the practical benefit of a unifying framework for assessing trainees and providing appropriate graduated responsibilities and supervision throughout training. It also discusses the importance of developing effective supervisor skills and ongoing faculty development to allow a shared mental model within faculty regarding the skills which a trainee should demonstrate at each level of their career.

Considerations for faculty developers: For faculty developers, this paper provides an effective overview of the interdependence of learner and supervisor, along with the impact this relationship has on graduated responsibility and patient care outcomes. Faculty developers are also provided with a framework that assists with the practical application of EPAs and assigning additional responsibilities within a clinical learning environment, as well as how to minimize areas of unwanted variability in assessment within a clinical environment. A construct is also provided for faculty developers on how a learner’s performance within an EPA can guide the identification of appropriate supervision levels, thereby promoting a balance between the supervision, autonomy, and advancement of trainees.

*3. Schultz K, Griffiths J, Lacasse M: The Application of Entrustable Professional Activities to Inform Competency Decisions in a Family Medicine Residency Program. Acad Med. 2015, 90:888-897 *[20]

Summary: This paper describes the process through which a Family Medicine residency program utilized EPAs to assess the development and proficiency of their residents. They faced a common challenge while transitioning to a competency-based assessment model in that their previous objectives did not integrate easily into the broader competencies. EPAs were found to be an effective tool to translate the broader competencies into specific activities that were easily observed and measured by preceptors and used for formative feedback. Experts from four Canadian Family Medicine programs formed a panel that first met to decide on the EPAs that would be used. Consideration was given to making sure a majority of patient presentations would fall into an EPA. The next steps involved designing a framework for the EPAs and the benchmarks within each activity. The clinical encounter was divided into eight phases: hypothesis formation, history, physical examination, investigation, diagnosis, treatment, follow-up, and referral. Activities were developed for each phase, along with graded levels of supervision, ranging from close, minimal, and ready for independence. The final step was the integration of the EPAs into an assessment system. Field notes were used for a daily formative assessment. Electronic notes allowed preceptors to select the level of supervision and then view each phase of the clinical encounter and the performance expected for that EPA, which helped preceptors develop a more objective view of their expectations for residents.

Relevance to junior faculty members: One of the challenges in assigning graduated responsibility to medical residents is the subjectivity of the judgment to provide independence. The framework used to create specific and measurable activities within each level of supervision could be adapted for various tasks and training levels within any residency. The paper also offers a particularly useful framework for junior faculty looking to systematically assess nearly any aspect of learner performance and gives examples of what performance at each of the levels of supervision would look like (close supervision, minimal supervision, and independent practice), a critical step toward appropriately assigning graduated responsibility. It also emphasizes the importance of different preceptors speaking the same language when providing feedback to learners or performing summative assessments as part of a Clinical Competency Committee (CCC).

Considerations for faculty developers:For faculty developers, this paper provides instruction for both creating specialty-specific EPAs, as well as integrating them into a new system of assessment for the purpose of assigning graduated responsibility. The manuscript describes how the EPAs were chosen, how they were incorporated into the existing assessment framework, and how EPA-specific templates were created. Faculty developers interested in creating EPAs and assigning graduated responsibility in this manner may benefit from this step-wise approach and plan for similar strategies. Another important consideration for faculty developers emphasized in this paper is to have a plan in place for “change management.” Integrating new EPAs into a competency-based assessment is no small task and will likely encounter resistance along the way. Faculty developers should plan to have sessions to inform stakeholders, receive feedback, and build trust and expertise to ensure buy-in. These sessions should include both faculty and residents, as both will be critical to the success of the new assessment program.

*4. Ten Cate O, Chen HC, Hoff RG, Peters H, Bok H, van der Schaaf M: Curriculum Development for the Workplace Using Entrustable Professional Activities (EPAs): AMEE Guide No. 99. Med Teach. 2015, 37:983-1002 *[21]

Summary: This manuscript summarizes the current literature on assigning clinical competency and is intended to serve as a guide for developing a competency-based curriculum incorporating EPAs. Competencies describe the qualities of the learner, while EPAs describe the work that is to be done in the workplace. Central to this idea is that competencies should be mapped to EPAs in a two-dimensional matrix. When viewed together in a matrix, it is possible to see what competencies must be obtained in order for a learner to be trusted to perform an EPA. Invariably, competencies map to multiple EPAs while the execution of an EPA requires multiple competencies. These matrices provide the trainee with an expectation of how to obtain trust to perform an EPA while providing the trainer with guidance on what to evaluate. The authors also present four questions that should be addressed during curriculum development: “What is the work to be done?” “What must trainees demonstrate before we can trust them to do the work?” “How should trainees be prepared to meet these requirements?” and “How do we assess trainees’ readiness to pass the threshold of entrustment?” In answering these questions, the manuscript focuses on identifying and validating EPAs, defining levels of supervision, detailing strategies for instructing trainees, and shifting our traditional assessment tools. The article encourages a change from linear scale or numerical assessment models to ones that focus on how much supervision is anticipated for the trainee when performing specific tasks. Finally, various assessment tools and instruments are provided that faculty developers can incorporate into their curriculum. 

Relevance to junior faculty members: This paper provides an excellent and comprehensive introduction to the world of EPAs, which form the basis of entrustment decisions and graduated responsibility for trainees to deliver care under a specific level of supervision. The authors highlight the clear distinction between competencies, milestones, and EPAs, while also suggesting how the concepts can be inter-related and used to appropriately assign responsibilities to learners who are ready. Key to this idea is the notion that entrustment decisions are typically informed by multiple competencies from several domains of practice. Armed with an understanding of the principles underlying EPA assessment, junior faculty may engage in more robust conversations about feedback and the formative and summative assessment of their trainees. 

Considerations for faculty developers: This manuscript serves as an excellent resource for faculty developers owing to both its scope and clarity. ten Cate and colleagues provide a relatively comprehensive guide to developing an EPA-based curriculum to assign graduated responsibility to residents, including tips for the practical application of information technology to aid in assessment. For those faculty developers hoping to overhaul assessment of residents in their training program, this effectively serves as a de-facto instruction manual. In particular, the authors do an excellent job at suggesting several methods for translating professional work into EPAs and offer commentary on a number of common pitfalls. Additionally, faculty developers will find advice about connecting milestones to competencies and EPAs particularly useful for moving toward an informed approach to assigning graduated responsibility to residents and a curriculum based on competency based-education. 

*5. Ten Cate O, Hart D, Ankel F, et al.: Entrustment Decision Making in Clinical Training. Acad Med. 2016, 91:191-198 *[22]

Summary: This key work by ten Cate discusses the entrustment decision-making process including the constructs of trust and entrustment in the workplace. The authors argue that granting autonomy at a designated level of supervision aligns more intuitively with current healthcare practice when compared to other methods of assessment. They distinguish different modes of trust and entrustment decisions. These models of trust represent a “readiness” judgment based on several factors. Ad hoc entrustment decisions by clinical supervisors about trainees are based on a mix of estimated trustworthiness of the trainee, risk of the situation, the urgency of the task, and suitability of the work at that moment for the trainee. Summative entrustment decisions are grounded in sufficient assessment data and made by CCCs for the trainee to act in the future with a specified level of supervision. These levels of supervision include observation, acting with direct supervision, acting with indirect supervision, acting without supervision, and providing supervision to other learners. This reflects how graduated responsibility is operationalized within a competency-based system. They also elaborate on five categories that encompass how supervisors make decisions to entrust trainees. These categories include factors related to the trainee, the supervisor, the situation or context, the task itself, and the relationship between the trainee and supervisor. Grounded, summative entrustment decisions are focused on factors related to the trainee only. These fundamentally include competence, conscientiousness, truthfulness, discernment of one’s limitations, empathy, interprofessional collaboration, self-confidence, self-directed reflection and improvement, sense of responsibility, and addressing mistakes. The assessment of these qualities requires longitudinal assessment across multiple contexts by multiple raters in order to generate decisions that can be trusted. 

Relevance to junior faculty members: Gradations of entrustment are essential to the process of graduated responsibility as it is tied directly into how much supervision a resident requires for each given task. This supervision and the level of autonomy changes over time with the provision of entrustment as trainees progress. Each faculty member forms ad hoc entrustment decisions each time he or she works with a resident. Being aware of the level of ad hoc entrustment, the supervision provided, and the results of those decisions will aid in directing future responsibility for each task. Each junior faculty member should keep in mind the five factors related to real-time decisions around entrustment: the trainee, the supervisor, the context, the task itself, and their interaction with the trainee. Each of these factors plays a significant role in when, how, and what tasks each trainee can be trusted with. By maintaining awareness of these frequently subconscious decisions, each faculty member can provide an appropriate level of supervision, ad hoc entrustment with targeted feedback, and valuable assessment data to the team formulating summative entrustment decisions for each trainee moving forward. 

Considerations for faculty developers: Faculty developers may use this manuscript as a basis for creating an infrastructure around both daily and long-term decisions regarding graduated responsibility and entrustable activities. Entrustment influences the level of supervision and independent responsibility offered by faculty. Specific training on ad hoc entrustment decisions may allow less knowledgeable faculty to make improved decisions about resident trustworthiness. The sources of information needed to make well-informed summative entrustment decisions discussed here will also be essential for faculty developers, as they will need to ensure assessment data is easy to obtain from faculty and access when used for summative assessment.

Limitations

We attempted to be as comprehensive as possible in our literature review, however, it is possible that relevant articles were missed, especially as CBME is a rapidly evolving field. Our Delphi group was composed of a select group of Emergency Medicine physicians from two countries. It is possible that a larger or more diverse panel would have made different choices about which articles to include, although it is important to note that none of the final five articles were specific to Emergency Medicine. Further, by utilizing junior faculty members in this panel, it is possible that a lack of experience allowed important topics to be missed, although we attempted to mitigate this by including more senior faculty as well. Importantly, the addition of junior faculty allows for a more diverse background and targeting of topics relevant to these end users [3]. Finally, this review is intended to identify key articles on the topic of graduated responsibility. It is not intended to serve as a comprehensive review of the entire topic, which would be beyond the scope of this endeavor.

## Conclusions

These five key articles on graduated responsibility provide a useful primer for junior faculty members interested in improving the state of graduated responsibility at their residency training program, including practical tips on entrustment decisions, entrustable professional activities, clinical supervision, competency decisions, and the role each plays in influencing graduated responsibility decisions. Our hope is that junior faculty can use the knowledge gained here to critically examine the training and assessment structures in place at their own residency and implement the improvements described here to create even better-graduated responsibility experiences for their residents.
